# Heart failure guideline implementation in developing countries: A testimony from Syria

**DOI:** 10.1002/ejhf.3625

**Published:** 2025-02-13

**Authors:** Amr Abdin, Mohammad Bashar Izzat, Ahmad Rasheed Alsaadi, Asim Katbeh, Yassin Bani Marjeh, Suleman Aktaa

**Affiliations:** ^1^ Internal Medicine Clinic III, Cardiology, Angiology and Intensive Care Medicine, Saarland University Hospital Homburg Germany; ^2^ Syrian Cardiovascular Association Damascus Syria; ^3^ Damascus University, Faculty of Medicine Damascus Syria; ^4^ Department of Cardiology St. Paul's Hospital Vancouver BC Canada

The European Society of Cardiology (ESC) develops clinical practice guidelines for a number of cardiovascular diseases including heart failure (HF) to provide evidence‐based, up‐to‐date recommendations designed to be applicable in daily practice.^1^, ^2^ These guidelines serve as a vital tool for healthcare professionals, offering guidance on patient management based on clinically reviewed contemporary evidence. However, observational studies highlight a gap and geographic variations in the implementation of guideline recommendations,^3^, ^4^ resulting in missed opportunities to reduce morbidity, mortality, and healthcare utilization associated with HF.^5^

The implementation of clinical practice guidelines in clinical practice is a complex and challenging process influenced by multiple factors. Numerous barriers and enablers have been identified.^6^ First, barriers related to the guidelines themselves include their complexity, limited accessibility, and poor applicability to real‐world practice. Second, barriers associated with healthcare providers include a lack of knowledge and skills, as well as language barriers in multi‐ethnic countries. Additionally, patient‐related factors, such as limited awareness, poor adherence, and financial constraints, play a significant role. Finally, institutional and resource‐related challenges, such as time constraints, suboptimal healthcare networks, inadequate interprofessional communication pathways, and insufficient incentives or reimbursement, further complicate effective implementation.

**Figure 1:**
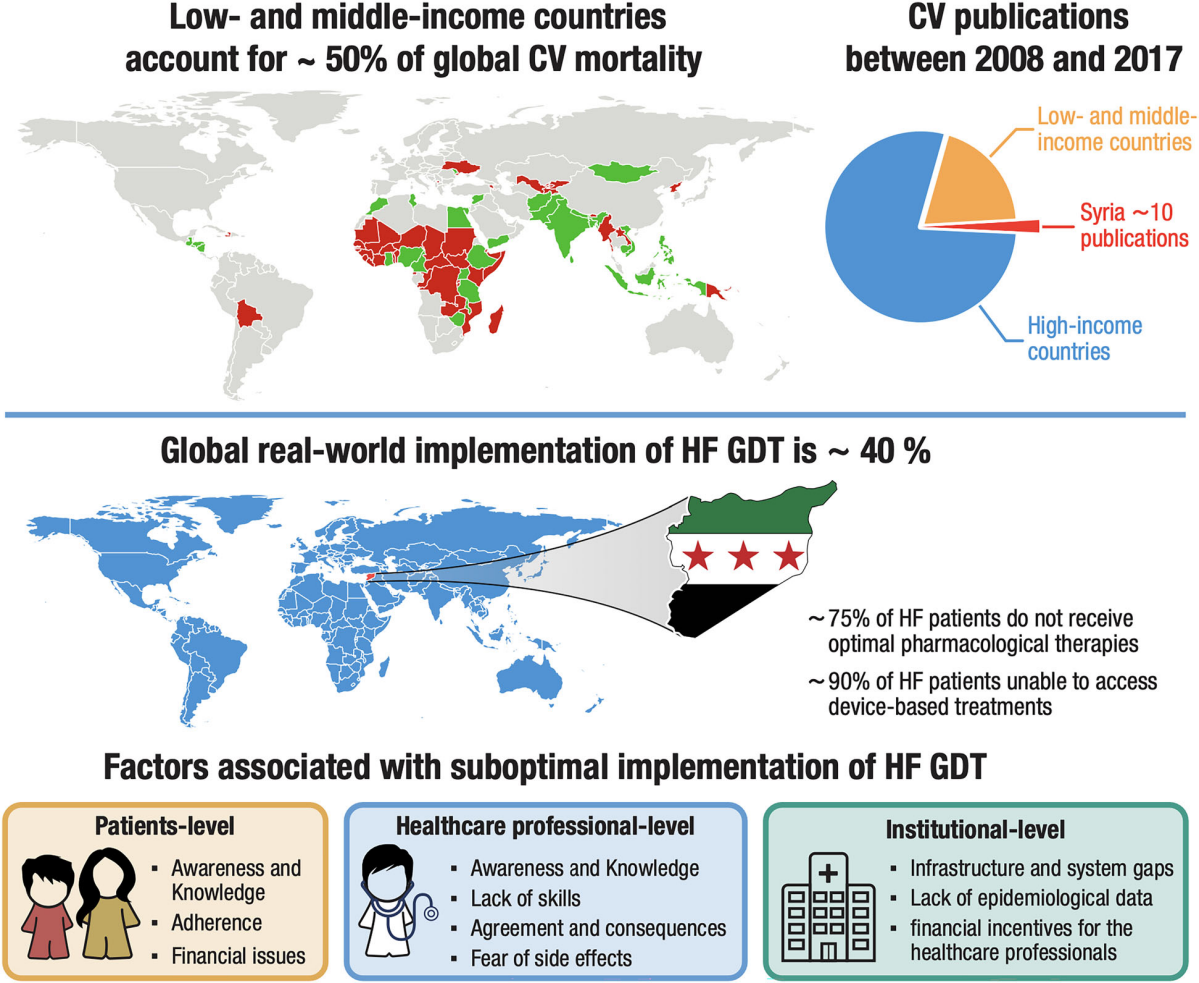
Real‐world implementation of guideline‐directed medical therapy (GDMT) in heart failure (HF) and factors associated with suboptimal prescription and use of GDMT in HF. CV, cardiovascular.

In many low‐ and middle‐income countries (LMICs), which account for approximately 50% of global cardiovascular mortality,^7^ the magnitude of the ‘evidence–practice’ gap in HF care is less appreciated and likely more substantial compared with developed countries (*Figure* *1*). Many developing countries lack a structured approach for HF care, with missed or delayed follow‐up and limited access to HF specialists and advanced HF therapies.^8^, ^9^ Understanding the healthcare systems and challenges in these regions is therefore critical. Unfortunately, research from LMICs remains sparse. For example, between 2008 and 2017, 80% of cardiovascular publications originated from high‐income countries, while only 0.2% came from LMICs. Syria, for instance, contributed approximately 5% of all publications from LMICs, which equates to just 10 cardiovascular disease publications over a decade.^7^, ^10^

The Syrian crisis had a profound impact on the Syrian healthcare system, resulting in the undertreatment of many cardiovascular conditions, including HF.^8^, ^11^ During the war years, Syria's healthcare infrastructure suffered extensive damage, leading to severe shortages of medical equipment, pharmacotherapies, and skilled healthcare professionals who are capable of performing complex and advanced procedures.^8^, ^12^ According to the Syrian Archive, more than 445 attacks on hospitals by the Syrian regime were documented, with the economic cost of the crisis exceeding 1 trillion euros.

In 2022, a survey was conducted in Syria to assess the implementation of recommended care processes for HF patients.^8^ The findings revealed that the ongoing economic crisis has placed an enormous strain on the Syrian healthcare system and patients alike, with over 70% of patients unable to access necessary treatments due to financial barriers, lack of local availability, and limited medical expertise. The survey showed that more than 50% of HF patients do not receive optimal guideline‐directed medical therapy (GDMT), and over 90% are unable to access advanced device‐based treatments due to their prohibitive costs. Furthermore, the absence of local expertise in performing cardiac resynchronization therapy (CRT) procedures adds another significant challenge to providing comprehensive care for HF patients in Syria (*Figure* *1*). According to the main and only local official companies providing devices in Syria over the last 10 years, fewer than five CRT devices were implanted in the entire country in both 2023 and 2024. This means that less than 1% of HF patients eligible for CRT received it.

Preventive measures for HF are also lacking in Syria, with poor optimization to long‐term illnesses, such as diabetes, hypertension, and chronic kidney disease. Surprisingly, to this day, the major hospitals in Syria lack facilities for primary percutaneous coronary intervention (PCI), and all acute myocardial infarction patients receive fibrinolysis instead. Primary PCI is only available in private settings. This is one of the most significant factors contributing to the development of HF.

Many individual approaches have been undertaken to address these gaps.^11^, ^12^ First, to share knowledge and raise awareness of comprehensive HF care across Syria, numerous educational meetings were organized in different cities. These efforts were led by physicians living abroad in collaboration with local physicians. Additionally, social media has been utilized as a vital platform to disseminate initiatives effectively.

Tailored approaches were also explored to adapt guideline recommendations to the Syrian financial and healthcare circumstances. For example, left bundle branch pacing (LBBP), which utilizes the traditional two‐chamber pacing system, but with the ventricular electrode implanted on the left bundle branch, was introduced as a viable and more affordable alternative treatment to biventricular CRT in patients with HF who have an indication for CRT. As part of this initiative, a number of LBBP cases were performed in Damascus through a collaborative effort between Syrian operators based in Germany and local Syrian physicians. The results were highly encouraging, demonstrating comparable outcomes to biventricular CRT while offering a cost‐effective solution for patients unable to afford biventricular CRT.^12^ Whilst the equipoise between LBBP and CRT is still awaiting a strong body of evidence, introducing LBBP in Syria was an extremely important initiative given the lack of availability, accessibility and affordability of CRT.

Now, with a renewed sense of motivation following the country's progress toward stability and freedom, we are committed to continuing this work with local physicians and look forward to expand such collaboration to healthcare authorities and decision‐makers to build on previous experiences and transform HF care across Syria to another level. Our focus is to initiate a comprehensive programme for HF care in Syria by defining the gaps in the current infrastructure and providing solutions that are both feasible and sustainable, such as the systematic collection of structured clinical data using harmonized definitions, as well as the participation in international clinical registries.

Such accomplishments can only be made possible through a dedicated support from international professional bodies such as the ESC and the World Health Organization. Strategies would be defined to spread knowledge among physicians in Syria at all levels, from medical students to cardiology consultants. This would be achieved by conducting educational meetings and workshops with the support of physicians from Europe and around the world. Additionally, efforts would be made to obtain educational grants from international bodies. Secondly, we aim to support hospitals with essential materials to improve patient outcomes, such as GDMT. Furthermore, we plan to establish programmes such as ‘Cardiac Devices for Syria’ to provide the country with pacemakers, implantable‐cardioverter defibrillators, and CRT devices, as these technologies are currently not widely available in Syria. Additionally, we aim to implement a programme called ‘PCI for Syria’ to provide primary PCI for myocardial infarction at the country's main hospital—an essential step in preventing HF. These goals can be achieved with the support of Western countries and international health organizations.
